# Osthole Inhibits Breast Cancer Progression through Upregulating Tumor Suppressor GNG7

**DOI:** 10.1155/2021/6610511

**Published:** 2021-02-27

**Authors:** Jie Mei, Tiejun Wang, Shaojie Zhao, Yan Zhang

**Affiliations:** ^1^Department of Gynecology and Obstetrics, Wuxi Maternal and Child Health Hospital, The Affiliated Hospital to Nanjing Medical University, Wuxi 214000, Jiangsu, China; ^2^Wuxi Clinical Medical College, Nanjing Medical University, Wuxi 214000, Jiangsu, China

## Abstract

Osthole (OST) is a plant-derived compound that can inhibit the proliferation of tumor cells and has a tumor-suppressive effect in multiple types of cancers. However, the mechanisms of OST-mediated breast cancer (BrCa) inhibition were still largely unknown. In this study, we made full use of the GSE85871 dataset to identify potential targets of OST in BrCa *via* multiple bioinformatics analysis. Next, a series of *in vitro* experiments were conducted to check the role of GNG7 in BrCa and the relationship between OST and GNG7. Through a series of bioinformatics analyses, GNG7 was identified as a potential target of OST, which could be significant upregulated by OST exposure in BrCa cells. Besides, GNG7 was lowly expressed in BrCa tissues compared with normal breast tissues, and BrCa patients with low GNG7 expression had shorter overall survival (OS) and relapse-free survival (RFS) compared with those with high GNG7 expression. Moreover, GNG7 silencing significantly enhanced cell proliferation and inhibited apoptosis, and exogenous overexpression of GNG7 showed reverse effects on BrCa cells. Last but not least, GNG7 inhibition could notably rescue OST-mediated cytotoxic effects. In summary, we identified GNG7 as a novel target for OST in BrCa and a potential tumor suppressor. Thus, OST could be therapeutically beneficial for BrCa through a GNG7-dependent mechanism.

## 1. Introduction

Breast cancer (BrCa) is a malignant tumor with the highest incidence in women worldwide [1, 2]. Since the late 1990s, the global incidence of BrCa has been on the rise. According to the latest statistics, the global incidence of breast cancer increased to 1,960,681 cases in 2017 [2]. Although China is not the country with a high incidence of BrCa, the current situation is still not optimistic. In recent years, the growth rate of BrCa in China is higher than that of European and American countries [3]. According to the latest statistics in China, the annual incidence of BrCa has reached approximately 272,400 cases [3]. There is no doubt that the prevention and treatment of BrCa have become a major public health problem. With the implementation of the “two-cancer” screening program for women and the progress of comprehensive treatment strategies, the mortality of BrCa has shown a downward trend. However, due to the complex pathogenesis of BrCa, the existing treatment methods still have some limitations.

**Figure 1:**
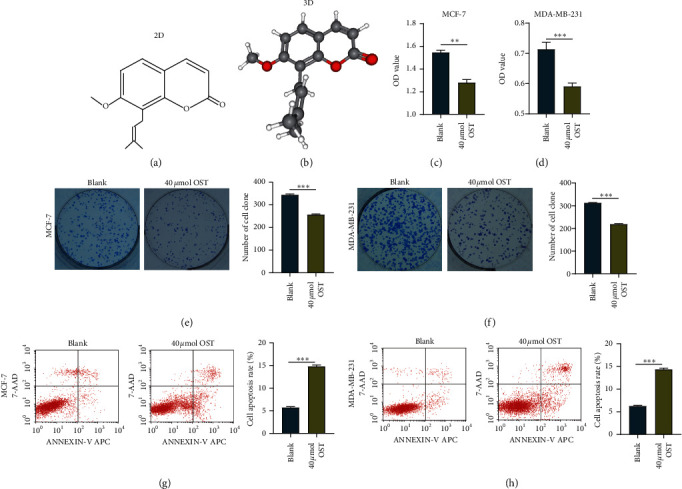
OST inhibits cell viability and induces apoptosis. (a, b) The 2- and 3-dimensional structure of OST. (c, d) The viability capacity of control and OST-treated BrCa cells was detected by CCK-8 assay. (e, f) The proliferative capacity of control and OST-treated BrCa cells was detected by clonogenic assay. (g, h) The apoptosis rate of control and OST-treated BrCa cells was detected by flow cytometry.

In the past few decades, more and more traditional Chinese medicines have been found to have anti-inflammatory and tumor suppression capabilities, which may open up new prospects for the treatment of malignant tumors [4, 5]. Osthole (OST, C_15_H_16_O_3_, molecular weight: 244.280 g/mol, Figures 1(a)–1(b)) is a plant-derived compound that can inhibit the proliferation of malignant cells and has an effective anticancer effect in multiple types of cancers [6]. OST has been shown to significantly inhibit the growth and metastasis of BrCa, thus serving as an effective candidate drug for the comprehensive treatment of BrCa [7, 8]. Although several studies have revealed the molecular mechanisms of OST in the treatment of BrCa [9], there is still a lack of systematic research on its molecular mechanism.

In this study, we downloaded the GSE85871 microarray dataset from the Gene Expression Omnibus (GEO) database, which includes the gene expression profiles of MCF-7 cells in the control group and the osthole-treated group. Through the systematic bioinformatics analysis, GNG7 was identified as a novel target of OST in BrCa. Then, a series of functional assays and analyses were performed to uncover the role of GNG7 in BrCa. In conclusion, the current research reveals that GNG7 acts as a novel tumor suppressor, which can be upregulated by OST in BrCa.

## 2. Materials and Methods

### 2.1. Reagents

Osthole (purity >99%, Catalog: O101699-5 g) was purchased from Aladdin Chemicals (Shanghai, China) and was dissolved in DMSO. Antibodies against GNG7 (Catalog: A10009), GAPDH (Catalog: AC002), and secondary antibodies were purchased from Abclonal (Wuhan, China). CCK-8 assay kit for cell proliferation detection (Catalog: KGA317) and Annexin V-PE/7-AAD double staining detection kit (Catalog: KGA1024) were purchased from KeyGEN BioTECH (Nanjing, China). Small interfering RNA (siRNA) for GNG7 inhibition and plasmid for GNG7 overexpression were synthesized by KeyGEN BioTECH (Nanjing, China). Sequences for siRNAs were as follows: siRNA-1 : GGAACAGCTACGCATAGAA; siRNA-2 : GTCAGCCACTAACAACATA; siRNA-3 : GAGCTACTGTGAGCAACAT.

### 2.2. GSE85871 Dataset Collection and Analysis

GSE40435 was a dataset including RNA-sequencing (RNA-seq) data of MCF-7 cells treated with DMSO and 102 traditional Chinese medicine contributed by Lv et al. [10]. The GSE40435 dataset was downloaded from the Gene Expression Omnibus (GEO) (https://www.ncbi.nlm.nih.gov/gds/) database, and RNA-seq data of the DMSO-treated control group (GSM2286316, GSM2286317) and the 10 *μ*mol osthole treatment group (GSM2286330, GSM2286331) was extracted for analysis. After probe filtering and probe merging, a total of 12,546 effective gene expression data were obtained. Next, “limma” R package was used to screen the differentially expressed genes (DEGs) between the control and the treatment group with the criteria of |log2 (FC)| ≥1 and *P* ≤ 0.05. Furthermore, the PPI network of DEGs was built by STRING database (https://string-db.org/) and visualized using Cytoscape software. Using cytohHubba plugin to screen hub genes in the PPI network, the 10 hub genes with the top 10 MCC scores on cytohHubba were further analyzed.

### 2.3. Cell Culture and Transfection

Human breast cancer cell lines (MDA-MB-231 and MCF-7) were obtained from the KeyGEN BioTECH (Nanjing, China). MDA-MB-231 and MCF-7 cells were cultured in RPMI-1640 and Leibovitz's L-15 medium (KeyGEN BioTECH, Nanjing, China) supplied with 5% CO_2_, respectively. Media in all cases were supplemented with 10% fetal bovine serum (Hyclone, Logan, UT), 100 units/mL penicillin, and 100 *μ*g/mL streptomycin. For subsequent functional assays, MCF-7 and MDA-MB-231 cells were transfected with GNG7-siRNA, GNG7 plasmid using Lipofectamine 2000 Reagent (Invitrogen, Carlsbad, USA) according to the manufacturer's instructions or exposed to OST.

### 2.4. GEPIA and Kaplan–Meier Plotter Analysis

GEPIA (http://gepia.cancer-pku.cn/) was an interactive website based on the Cancer Genome Atlas (TCGA) database [11]. In the current study, the GEPIA website was used to explore the expression level of GNG7 in BrCa and adjacent breast tissue samples. Besides, Kaplan–Meier plotter (http://kmplot.com/analysis) database was used to evaluate the prognostic value of GNG7 expression for overall survival (OS) and relapse-free survival (RFS) in BrCa patients [12]. BrCa samples were split into low expression group and high expression group depending on the median of the GNG7 expression level.

### 2.5. Linked Omics Analysis

Linked Omics (http://www.linkedomics.org/login.php) is a web database comprising multiomics datasets for analysis of 32 TCGA cancer types [13]. In this research, the Pearson correlation coefficient was used to statistically analyze the genes coexpressed with GNG7 with the threshold value of Pearson *R* >0.3. As a result, a total of 722 and 193 genes positively and negatively correlated with GNG7 expression were extracted.

### 2.6. Quantitative Real-Time PCR (qRT-PCR)

Total RNA of BrCa cells was extracted using TRIzol reagent (Invitrogen, Carlsbad, USA). The primers for GNG7 mRNA reverse transcription were synthesized in KeyGEN BioTECH (Nanjing, China). qRT-PCR was conducted using the One-Step TB GreenTM PrimeScriptTM RT-PCR Kit II (SYBR Green, TaKaRa, Japan). The 2^−ΔΔCt^ method was used for mRNA expression analysis. Primers used for gene amplification were as follows: GNG7: 5′ TGGTGGAACAGCTACGCATAGA 3′ (forward), 5′ CGGGCATGTTGCTCACAGTAG 3′ (reverse); GAPDH: 5′ AGATCATCAGCAATGCCTCCT 3′ (forward), 5′ TGAGTCCTTCCACGATACCAA 3′ (reverse).

### 2.7. Western Blot Analysis

Cells were placed in 35 mm dishes (6 × 10^5^ cells/dish). After transfection or exposure to OST, total proteins were harvested with lysis buffer. SDS-polyacrylamide gel electrophoresis and Western blotting analysis were performed as standard protocols. The primary antibodies for GNG7 (1 : 500 dilution, Catalog: A10009, Abclonal) and GAPDH (1 : 5000 dilution, Catalog: AC002, Abclonal) were used. Expression levels of proteins were normalized to GAPDH for each sample.

### 2.8. Cell Viability Assay

Cell viability was measured through CCK-8 assay. After transfection or exposure to OST, BrCa cells were seeded in 96-well tissue culture plates at a density of 4 × 10^4^ cells/ml (100 *μ*l/well) and fostered at 37°C in a constant-temperature incubator with 5% CO_2_ for 48 h. To each well, 10 *μ*l CCK-8 was added, after which the plate was put in the incubator for 2 h. The OD value of each well was measured at 450 nm by a microplate reader. Each experiment was repeated three times.

### 2.9. Apoptosis Analysis

Cells were plated in 60 mm dishes and allowed to attach overnight. Cells were transfected with GNG7-siRNA and GNG7 plasmid or exposed to OST. Following treatments, cells were fixed then labeled with Annexin V-PE/7-AAD (for apoptosis detection). Analysis was performed using FACSCalibur flow cytometer. Data for apoptosis was analyzed using FlowJo7.6 software.

### 2.10. Statistical Analysis

All statistical analyses were calculated using SPSS 26.0 software (Chicago, IL). Most of the data were analyzed by Student's *t*-test or one-way ANOVA followed by Dunnett's multiple posthoc tests. All data are presented as means ± SDs of five independent experiments if not noted. The prognostic value of GNG7 expression for OS and RFS in BrCa patients was checked by log-rank test. All statistical tests were two-sided, and *P* value ≤0.05 was considered statistically significant.

## 3. Results

### 3.1. Systematic Bioinformatics Analysis Reveals GNG7 as a Novel Target of OST

**Figure 2:**
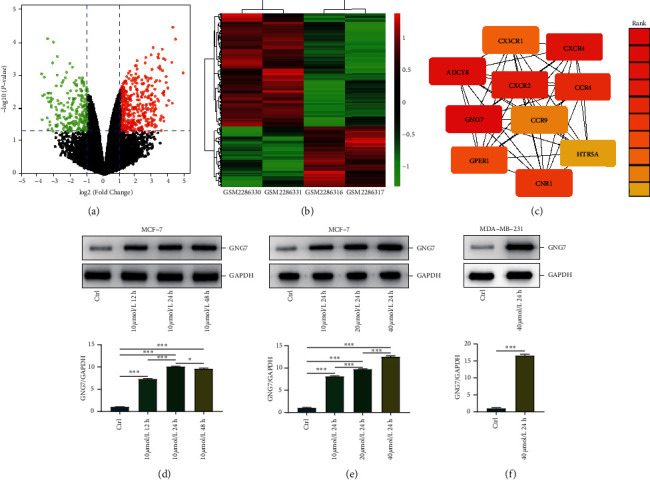
Identification of GNG7 as a novel target of OST in BrCa. (a, b) Volcano map and heatmap of DEGs between control and OST-treated MCF-7 cells. (c) Hub genes screening and MCC scores in PPI network. (d) OST upregulated GNG7 in a time-dependent manner in MCF-7 cells. (e) OST upregulated GNG7 in a dose-dependent manner in MCF-7 cells. (f) OST upregulated GNG7 in MDA-MB-231 cells treated with 40 *μ*mol for 24 h.

An increasing number of studies revealed that OST acted as an inhibitory role in the oncogenesis and development of BrCa [7–9]; there is still a lack of systematic research on its molecular mechanisms. To obtain a comprehensive insight into its molecular mechanisms, we use the “limma” R package to extract DEGs between the control and the treatment group. The results showed that 697 DEGs were satisfying the criterion of |log2 (FC)| ≥1 and *P* ≤ 0.05, among which 453 genes were upregulated and 244 genes were downregulated (Figure 2(a)). Heatmap analysis showed that DEGs had significant expression differences between the two groups (Figure 2(b)). In order to further explore the functions of DEGs and screen the key genes, we used the STRING database to construct a PPI network for 697 DEGs, and the results are shown in Figure S1. Then, we use the MCC algorithm to select GNG7 for further analysis with the highest score in the PPI network (Figure 2(c)). We next checked the impact of OST exposure on GNG7 expression. As shown in Figure 2(d), in MCF-7 BrCa cells, GNG7 was maximally upregulated by exposure to OST with 24 h, and with the increase of dose, OST increased GNG7 expression in a dose-dependent manner (Figure 2(e)). Furthermore, GNG7 was also significantly upregulated in MDA-MB-231 cells by OST treated with 40 *μ*mol for 24 h (Figure 2(f)). Overall, these findings suggest GNG7 is a novel target of OST in BrCa.

### 3.2. OST Suppresses Viability and Induces Apoptosis in BrCa Cells

To validate the inhibitory role of OST (40 *μ*mol) in BrCa cells, we next assessed the viability of MCF-7 and MDA-MB-231 cells following exposure to OST by CCK-8 assay. Our results exhibited that exposure of these BrCa cells to OST reduced cell viability (Figures 1(c)–1(d)). In addition, the clonogenic assay also validated the inhibitory function of OST in cell proliferation (Figures 1(e)–1(f)). Reduced viability in BrCa cells following OST exposure prompted us to determine whether OST could induce cell apoptosis. Staining of cells with annexin V/PI showed induction of cellular apoptosis in MCF-7 and MDA-MB-231 cells after osthole exposure (Figures 1(g)–1(h)). Taken together, OST has a notable tumor-suppressive effect on BrCa cells at a concentration of 40 *μ*mol.

### 3.3. Expression and Potential Functions of GNG7 in BrCa

**Figure 3:**
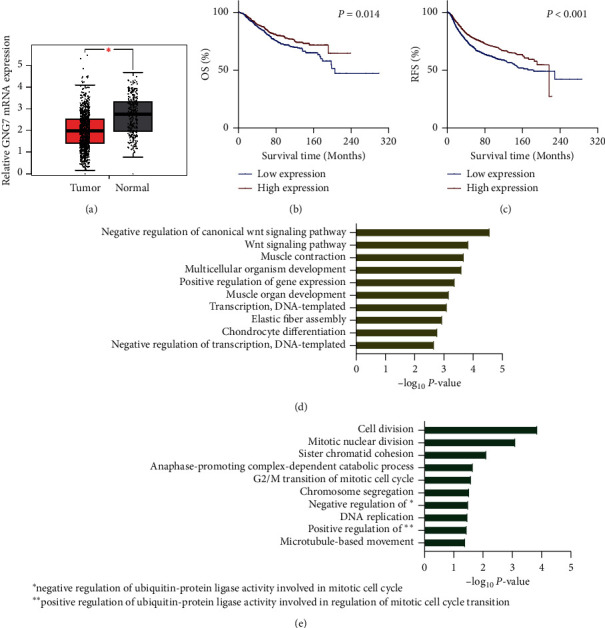
Expression and enrichment analysis of GNG7 in BrCa. (a) GNG7 was lowly expressed in BrCa tissues compared with normal tissues. (b, c) Low GNG7 expression was associated with poor OS and RFS in BrCa patients. (d) Biological process analysis of genes that positively correlated with GNG7. (e) Biological process analysis of genes that negatively correlated with GNG7.

Given that the role of GNG7 in BrCa has not been uncovered, we next determined the expression and potential functions of GNG7. GEPIA analysis revealed that GNG7 was lowly expressed in BrCa tissues compared with normal breast tissues (Figure 3(a)). Survival analysis exhibited that BrCa patients with low GNG7 expression had shorter OS and RFS compared with those with high GNG7 expression (Figures 3(b) and 3(c)). Furthermore, based on the TCGA data, we performed correlation and enrichment analysis. A total of 722 and 193 genes were positively and negatively correlated with GNG7 expression in the TCGA dataset (*R* >0.3). Besides, enrichment analysis revealed that genes positively correlated with GNG7 mainly enriched in negative regulation of canonical Wnt signaling pathway, and genes negatively correlated with GNG7 primarily regulated cell division (Figures 3(d) and 3(e)). Overall, these results indicate that GNG7 is a potential tumor suppressor that is associated with multiple functional biological processes in BrCa.

### 3.4. GNG7 Acts as a Tumor Suppressor in Regulating Cell Viability and Apoptosis

**Figure 4:**
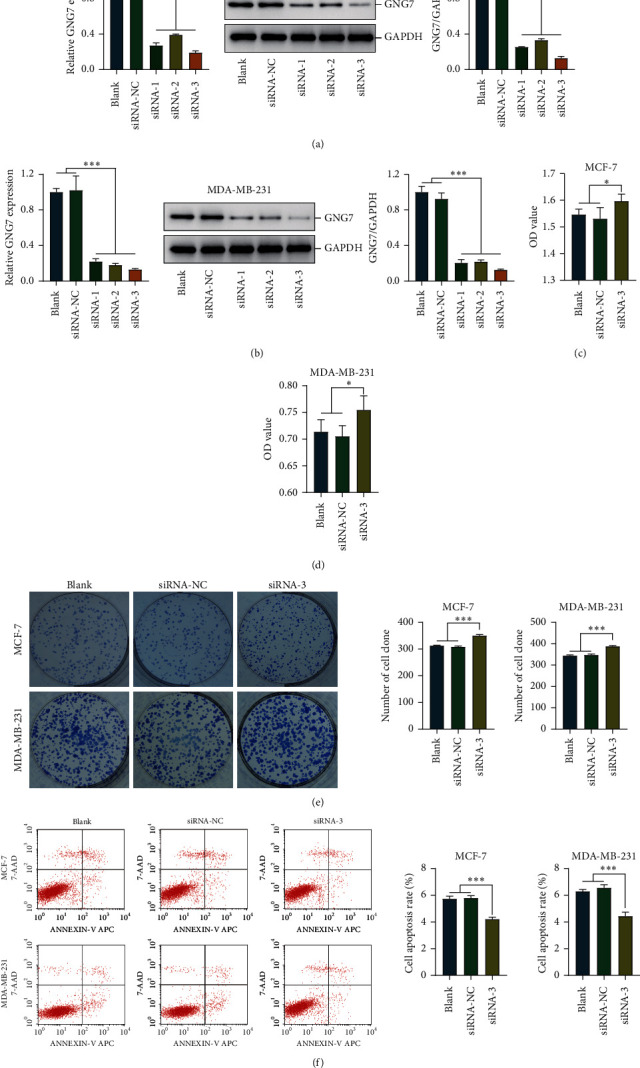
Silencing of GNG7 promotes cell proliferation and induces apoptosis. (a, b) The silencing efficiency of GNG7 in BrCa cells was confirmed by qPCR and western blotting. (c, d) The viability capacity of control and GNG7-silencing BrCa cells was detected by CCK-8 assay. (e) The proliferative capacity of control and GNG7-silencing BrCa cells was detected by clonogenic assay. (f) The apoptosis rate of control and GNG7-silencing BrCa cells was detected by flow cytometry. Note: ^*∗*^*P* < 0.05, ^*∗∗*^*P* < 0.01, ^*∗∗∗*^*P* < 0.001.

SiRNA-mediated silencing of GNG7 expression in the MCF-7 and MDA-MB-231 BrCa cells was performed to assess the functional role of GNG7 in BrCa in vitro. The functional role of GNG7 on cell proliferation and apoptosis in BrCa cells was tested by CCK-8, clonogenic assay, and flow cytometry, respectively. Firstly, the silencing efficiency of GNG7 in BrCa cells was confirmed by qPCR and western blotting (Figures 4(a) and 4(b)). Compared with the control cells, MCF-7 and MDA-MB-231 cells with GNG7 silencing showed enhanced proliferative capacity (Figures 4(c) and 4(e)). Besides, GNG7-siRNA significantly inhibited cell apoptosis in these two cell lines (Figure 4(f)).

**Figure 5:**
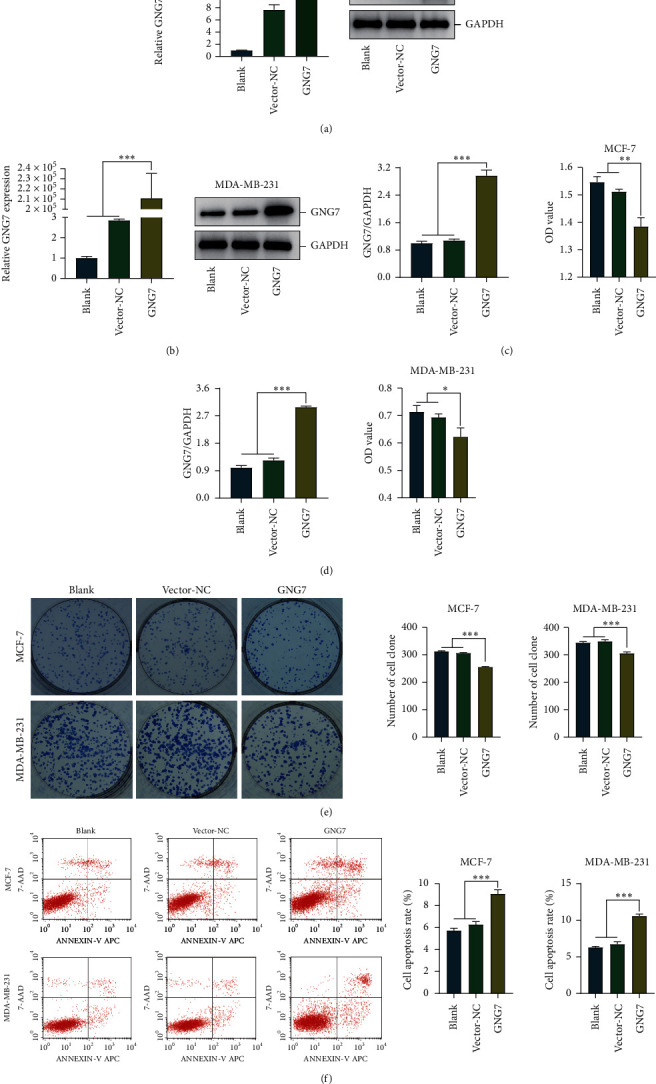
GNG7 overexpression inhibits cell proliferation and promotes apoptosis. (a, b) The transfection efficiency of GNG7 in BrCa cells was confirmed by qPCR and western blotting. (c, d) The viability capacity of control and GNG7-overexpressed BrCa cells was detected by CCK-8 assay. (e) The proliferative capacity of control and GNG7-overexpressed BrCa cells was detected by clonogenic assay. (f) The apoptosis rate of control and GNG7-overexpressed BrCa cells was detected by flow cytometry. Note: ^*∗*^*P* < 0.05, ^*∗∗*^*P* < 0.01, ^*∗∗∗*^*P* < 0.001.

Next, we assessed the change of cellular behaviors after exogenous GNG7 overexpression. Transfection efficiency of GNG7 in MCF-7 and MDA-MB-231 BrCa cells and was confirmed by qPCR and western blotting (Figures 5(a) and 5(b)). Compared with the control cells, the proliferative capacity of GNG7-overexpressed MCF-7 and MDA-MB-231 cells were significantly inhibited (Figures 5(c)–5(e)). In addition, exogenous expression of GNG7 remarkably induced BrCa cell apoptosis (Figure 5(f)). Overall, GNG7 acts as a novel tumor suppressor in BrCa, which may be a potential target for BrCa therapy.

### 3.5. GNG7 Inhibition Rescued OST-Mediated Cytotoxic Effects

**Figure 6:**
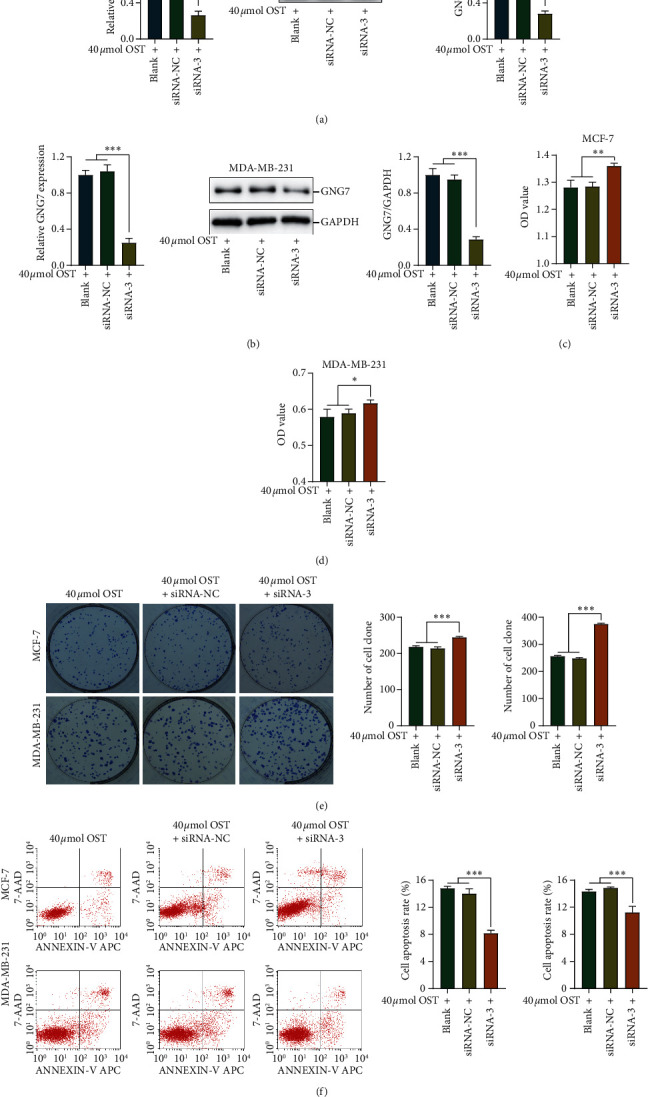
GNG7 inhibition rescued OST-mediated cytotoxic effects. (a, b) The expression of GNG7 in BrCa cells was confirmed by qPCR and western blotting. (c, d) The viability capacity of BrCa cells in the OST-treated, OST-treated + siRNA-NC, and OST-treated + siRNA-GNG7 group was detected by CCK-8 assay. (e) The proliferative capacity of BrCa cells in the OST-treated, OST-treated + siRNA-NC, and OST-treated + siRNA-GNG7 groups was detected by clonogenic assay. (f) The apoptosis rate of BrCa cells in the OST-treated, OST-treated + siRNA-NC, and OST-treated + siRNA-GNG7 groups was detected by flow cytometry. Note: ^*∗*^*P* < 0.05, ^*∗∗*^*P* < 0.01, ^*∗∗∗*^*P* < 0.001.

We next confirmed the involvement of GNG7 in OST-induced cytotoxic effects by silencing GNG7. First of all, the expression levels of GNG7 in OST-treated, OST-treated + siRNA-NC, and OST-treated + siRNA-GNG7 groups were checked by qPCR and western blotting (Figures 6(a) and 6(b)). Silencing of GNG7 expression was utilized to enhance OST-induced inhibition of cell proliferation (Figures 6(c)–6(e)). Besides, GNG7 knockdown also reversed OST-induced cell apoptosis (Figure 6(f)). To sum up, these results suggest that the inhibitory activity of OST in BrCa cells is, at least partly, mediated through the upregulation of GNG7.

## 4. Discussion

The antitumor effects of OST have been confirmed in many types of tumors, such as BrCa [7], gastric cancer [14], ovarian cancer [15], cervical cancer [16], and nasopharyngeal cancer [17]. In most research, OST was dissolved in DMSO, and OST showed antitumor effect in the concentration range of 20 *μ*mol–200 *μ*mol in BrCa [7, 8, 18]. A few studies have explored the antitumor mechanism of OST in the treatment of BrCa. For example, Hung et al. demonstrated that OST can inhibit the hepatocyte growth factor- (HGF-) induced epithelial-mesenchymal transition (EMT) of BrCa cells by inhibiting the c-Met/Akt/mTOR pathway [19]. However, there is no systematic study to explore the antitumor mechanisms of OST in BrCa. In recent years, the development of microarray and RNA-sequencing technology has greatly promoted the exploration of the function and mechanism of traditional Chinese medicines [20, 21].

In this study, we made full use of the GEO database to identify potential targets of OST in BrCa through a comprehensive analysis of the GSE85871 dataset. Through the differential gene analysis, we obtained a total of 697 DGEs. Besides, through PPI network construction and key genes screening, we extracted several hub genes, of which GNG7 showed the strongest interaction score. In this study, we found that GNG7 was significantly upregulated after OST exposure, and GNG7 inhibition rescued OST-mediated cytotoxic effects. Totally, GNG7 upregulation may be one of the key mechanisms for OST-induced suppression of BrCa progression.

GNG7 is G protein *γ* subunit 7, and its low expression is considered to be a key event in the occurrence of malignant tumors [22]. In oesophageal cancer, GNG7 was lowly expressed in tumor tissues and low expression of GNG7 was significantly associated with poor prognosis, which could be used as a potential biomarker for cancer diagnosis and prognostic evaluation [23]. In terms of molecular mechanisms, GNG7 can induce autophagy and affect cell division by destroying the actin skeleton [24]. Besides, GNG7 silencing promoted the proliferation and differentiation of placental cytotrophoblasts in preeclampsia rats *via* activating the mTOR signaling pathway [25]. However, the role of GNG7 in BrCa has not been defined, which may be further explored in the future.

In the current study, the RNA-seq data in BrCa tissues from the GEPIA database was used to analyze the differential expression of GNG7 in BrCa and normal breast tissues. GEPIA analysis suggested that GNG7 expression was a loss in BrCa tissues compared with normal breast tissues. Besides, survival analysis from the Kaplan–Meier plotter exhibited that BrCa patients with low GNG7 expression had shorter OS and RFS compared with those with high GNG7 expression. Moreover, correlation and enrichment analysis uncovered that GNG7 might negatively regulate the canonical Wnt signaling pathway and cell division. In addition, we confirmed that targeting GNG7 expression using siRNA significantly enhanced cell proliferation and inhibited apoptosis. However, exogenous overexpression of GNG7 showed reverse effects on MCF-7 and MDA-MB-231 cells.

## 5. Conclusion

In conclusion, we, for the first time, identified GNG7 as a novel target for OST in BrCa; in other words, OST could be therapeutically beneficial for BrCa through a GNG7-dependent mechanism. Besides, we also explored the function of GNG7 on BrCa cells and uncovered GNG7 as a tumor suppressor in BrCa, which may be a potential target for BrCa therapy.

## Data Availability

All the data used during the study are available from the corresponding author on request.

## Supplementary Materials

Supplementary Materials Figure S1: construction of PPI network by STRING database.
