# Supplementary figures and images for: Osthole Inhibits Breast Cancer Progression through Upregulating Tumor Suppressor GNG7
Source: J Oncol. 2021 Feb 27;2021:6610511. doi: 10.1155/2021/6610511 (PMC7937475; doi:10.1155/2021/6610511)

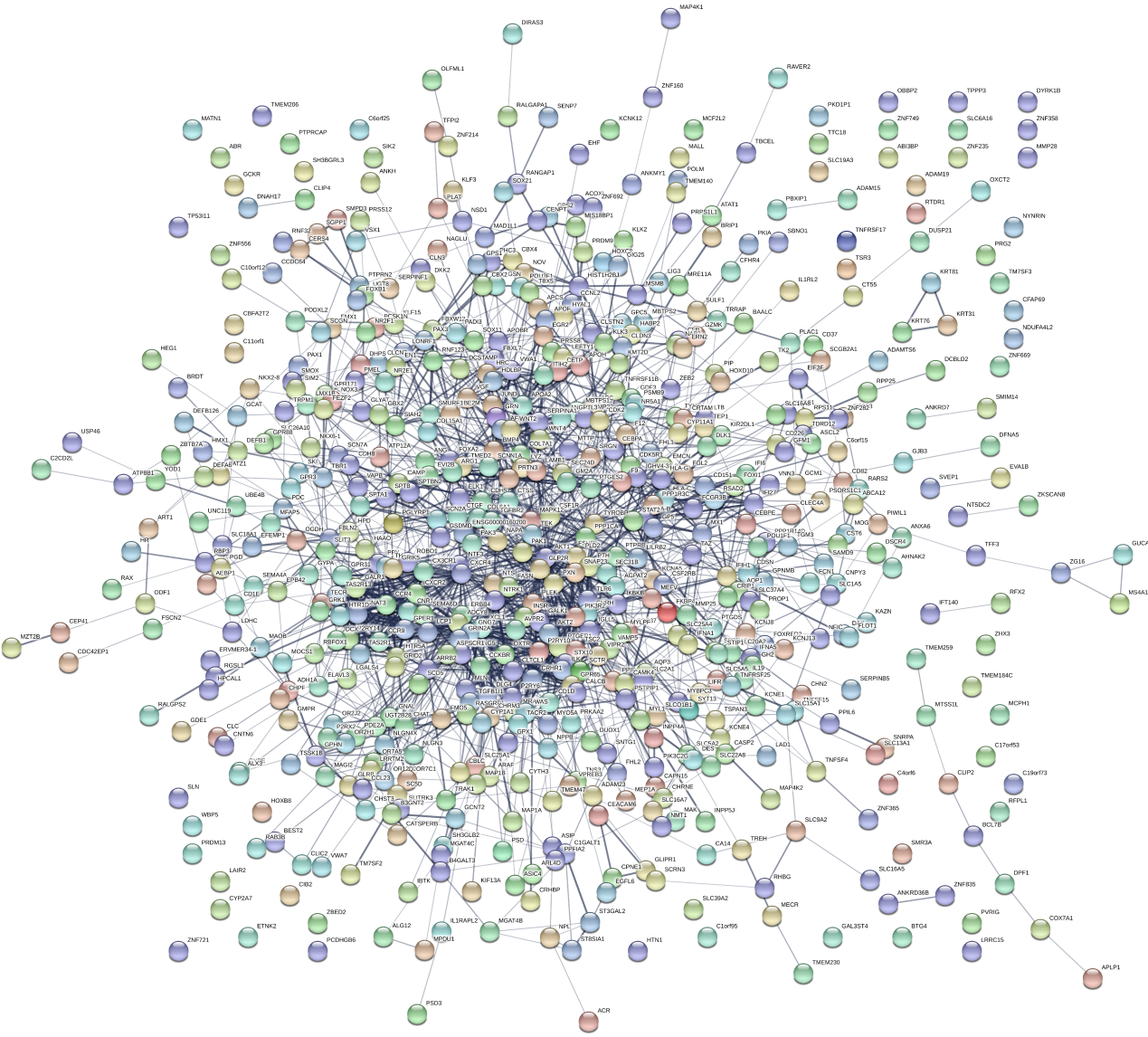


Figure S1. Construction of PPI network by STRING database.

Supplement: Supplementary Materials — Figure S1: construction of PPI network by STRING database. [file 6610511.f1.docx]
